# Case Report: Syphilitic proximal coronary stenosis with discordant serology: diagnostic validation by optical coherence tomography

**DOI:** 10.3389/fmed.2025.1714726

**Published:** 2025-11-11

**Authors:** Jingjie Xiong, Ting Gan, Jing Hu, Wenhu Liu, Xuehua Wang, Han Li, Jiaxi Lv, Ni Xiong, Yanli Huang, Qianyi Liu, Lihui Yin, Yan Wang, Zhaohui Wang, Ya Wang

**Affiliations:** 1 Department of Cardiology, Union Hospital, Tongji Medical College, Huazhong University of Science and Technology, Wuhan, China; 2 Hubei Key Laboratory of Biological Targeted Therapy, Union Hospital, Tongji Medical College, Huazhong University of Science and Technology, Wuhan, China; 3 Hubei Provincial Engineering Research Center for Immunological Diagnosis and Therapy for Cardiovascular Diseases, Union Hospital, Tongji Medical College, Huazhong University of Science and Technology, Wuhan, China; 4 Department of Cardiology, Beijing Anzhen Hospital, Capital Medical University, Beijing, China; 5 Department of Infectious Diseases, Union Hospital, Tongji Medical College, Huazhong University of Science and Technology, Wuhan, China

**Keywords:** syphilitic vasculitis, aortitis, optical coherence tomography, intravascular imaging, proximal coronary stenosis, discordant serology, Treponema pallidum

## Abstract

Cardiovascular syphilis, a late manifestation of *Treponema pallidum* infection, remains a diagnostic challenge, particularly when serological tests are discordant. We report a case of a 70-year-old male with recurrent chest tightness and discordant syphilis serology (non-reactive RPR, reactive TPPA). Coronary angiography revealed a critical stenosis at the distal left main coronary artery, specifically involving the ostio-proximal segment of the left anterior descending (LAD) artery. Critically, the absence of systemic atherosclerosis was evidenced by a coronary artery calcium score of zero and normal carotid intima-media thickness. Optical coherence tomography (OCT) provided definitive diagnostic clarity by revealing microstructural features pathognomonic for syphilitic vasculitis: vasa vasorum obliteration, elastic lamina fragmentation, and adventitial fibrosis with microcalcifications. These findings were distinct from atherosclerotic plaque. The patient successfully underwent OCT-guided percutaneous coronary intervention followed by targeted antibiotic therapy. This case highlights the indispensable role of OCT in establishing the etiology of non-atherosclerotic proximal coronary stenosis, underscoring that cardiovascular syphilis must be considered even with non-reactive non-treponemal tests in late-stage disease.

## Introduction

Syphilis, caused by the spirochete *Treponema pallidum*, remains a substantial global health burden with an estimated 7.1 million annual incident cases (1). Despite antibiotic availability, its tertiary-stage complications, particularly cardiovascular manifestations, continue to pose significant diagnostic challenges. Cardiovascular involvement is a classic late manifestation that typically occurs 10–30 years after infection. In the pre-antibiotic era, approximately 5–10% of untreated infections progressed to cardiovascular syphilis; in contemporary practice, with widespread screening and treatment, it has become uncommon. Syphilitic aortitis, the hallmark lesion, primarily affects the proximal aorta and may lead to complications such as aortic aneurysm, aortic regurgitation, and ostial or proximal coronary artery stenosis due to the direct extension of the inflammatory process. This pathophysiologic pattern underscores the importance of considering syphilis in patients presenting with these specific cardiovascular pathologies, even when serological findings are atypical. A key difficulty is that up to 28% of tertiary syphilis cases exhibit a non-reactive rapid plasma reagin (RPR) test despite a reactive treponemal test, often leading to delayed diagnosis. These diagnostic difficulties are compounded by nonspecific clinical presentations mimicking idiopathic aortitis or atherosclerosis, limitations of conventional imaging in detecting early microvascular changes, and the absence of pathognomonic biomarkers (2). We present an illustrative case of RPR-negative cardiovascular syphilis definitively diagnosed through OCT, revealing three characteristic microarchitectural signatures: vasa vasorum obliteration, elastic laminae fragmentation, and adventitial microcalcifications, thereby establishing OCT-derived vascular histomorphology as a critical diagnostic tool when serological tests prove inconclusive in late-stage disease (3).

## Case report

**Figure 1:**
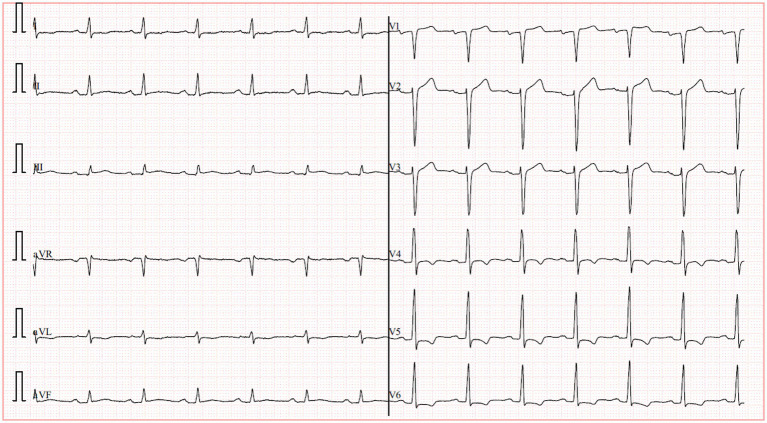
Pathological Q waves in leads V1–V3.

**Figure 2:**
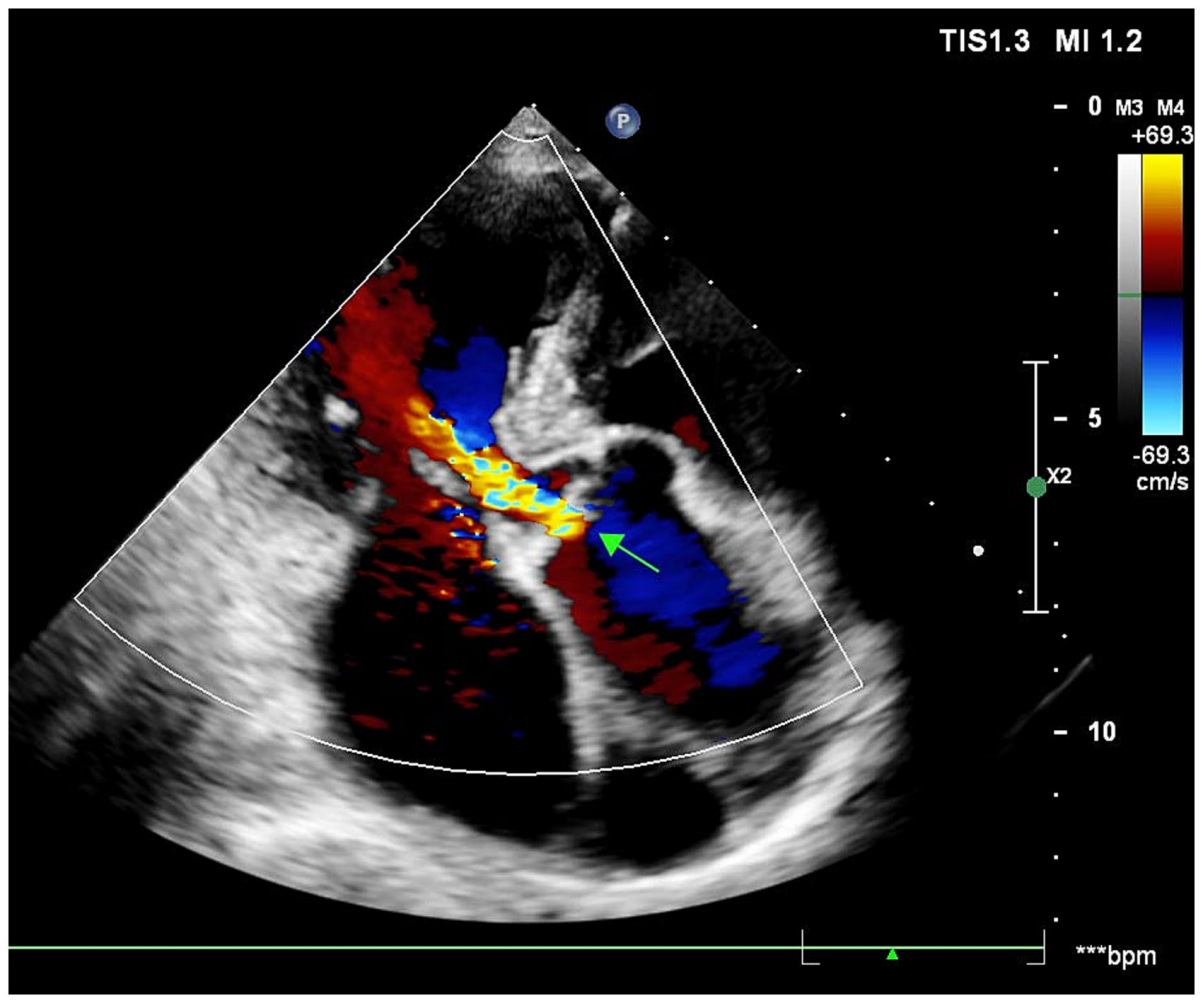
Echocardiographic features of aortic valvulopathy.

A 70-year-old male presented with recurrent substernal chest tightness, described as pressure-like, persisting for 1 week prior to admission. His past medical history included hospitalization for unexplained chest tightness 5 years ago. He also disclosed multiple unprotected sexual partners in his youth. He was a lifetime non-smoker, had no history of hypertension or diabetes mellitus, and his body mass index was 23.5 kg/m^2^. Electrocardiogram showed normal findings. The patient denied any family history of hypertension or cardiovascular disorders. Upon admission, vital signs were within normal limits, demonstrating a regular heart rate of 78 bpm and normotensive blood pressure (110/70 mmHg). Physical examination revealed diminished breath sounds bilaterally, with cardiomegaly noted in the left lower chest. Cardiac auscultation demonstrated a diminished, forceful apex beat and a grade 3/6 systolic blowing murmur at the apical region. The abdomen was soft and non-tender, without hepatosplenomegaly, rebound tenderness, or guarding; Murphy’s sign was negative. No lower extremity edema was present. High-sensitivity C-reactive protein (hs-CRP) was 2.4 mg/L. Mild dyslipidemia was noted with a low-density lipoprotein cholesterol (LDL-C) level of 1.76 mmol/L and a high density lipoprotein cholesterol (HDL-C) level of 0.81 mmol/L. Cardiac troponin I was 2.26 ng/dL and creatinine kinase myocardial band was 27.6 U/L. Serological testing revealed a non-reactive RPR (titer 1:1) but a positive *Treponema pallidum* particle agglutination (TPPA), confirming discordant serology. The initial electrocardiogram was unremarkable. However, a subsequent ECG revealed left ventricular hypertrophy (LVH) with ST-T changes in collateral leads and pathological Q waves in leads V1–V3 (Figure 1). The chest radiograph demonstrated mediastinal widening consistent with aortic dilation. A non-contrast cardiac computed tomography (CT) scan was performed to assess the aorta and coronary calcification, which revealed a thoracic aortic aneurysm (diameter 5.2 cm) but, remarkably, a coronary artery calcium score (CACS) of 0, indicating the absence of any calcified atherosclerotic plaque. Echocardiography revealed significant cardiac abnormalities including left atrial and ventricular enlargement with phasic left ventricular wall motion abnormalities, mild aortic valve thickening accompanied by moderate diastolic regurgitation, and severe mitral regurgitation. The study also demonstrated grade III left ventricular diastolic dysfunction and markedly reduced systolic function with an ejection fraction of 29%. Collectively, these findings suggested secondary mild pulmonary hypertension in the setting of significant left heart disease (Figure 2).

**Figure 3:**
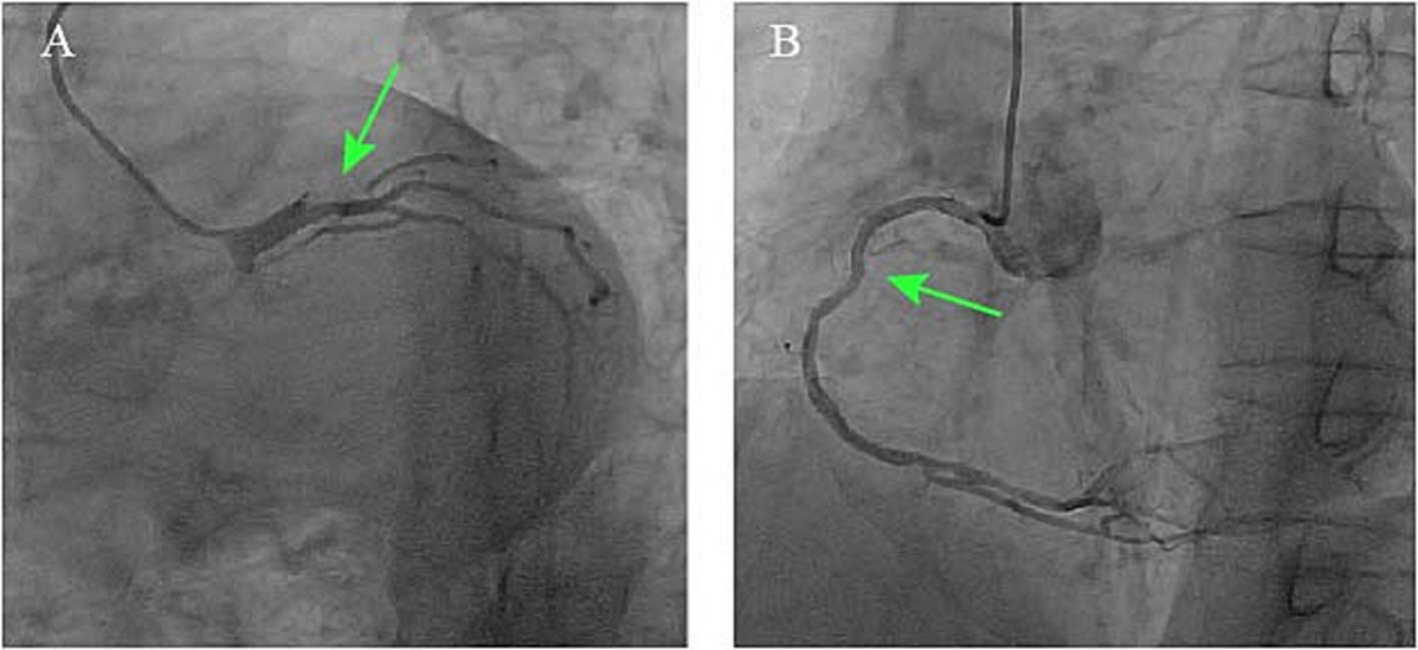
Coronary angiograms demonstrating **(A)** critical stenosis at the distal left main coronary artery involving the LAD ostio-proximal segment (green arrow) and **(B)** right coronary artery diffuse disease with Rentrop grade 2 collaterals.

Given the patient’s indeterminate symptom onset timeline, absence of acute anginal symptoms, and hemodynamic stability, guideline-directed medical therapy for heart failure was initiated, comprising antiplatelet therapy, statins, beta-blockers, angiotensin-converting enzyme inhibitors, and mineralocorticoid receptor antagonists. Antibiotic treatment was deferred until completion of the diagnostic workup. After 10 days of medical management, the patient had no significant symptoms of heart failure. A follow-up echocardiogram after 10 days of medical therapy showed persistent severe left ventricular dysfunction (EF 29%) and unchanged valvular abnormalities. Selective coronary angiography demonstrated a critical stenosis at the distal left main coronary artery, specifically involving the ostio-proximal segment of the LAD artery with diffuse disease involving the right coronary artery. Notably, grade 2 intercoronary collateral circulation (Rentrop classification) was observed between the right and left coronary systems (Figure 3). To further evaluate systemic atherosclerotic burden, a carotid artery ultrasound was performed, revealing a normal intima-media thickness (CIMT) of 0.7 mm bilaterally, with no evidence of plaque formation.

**Figure 4:**
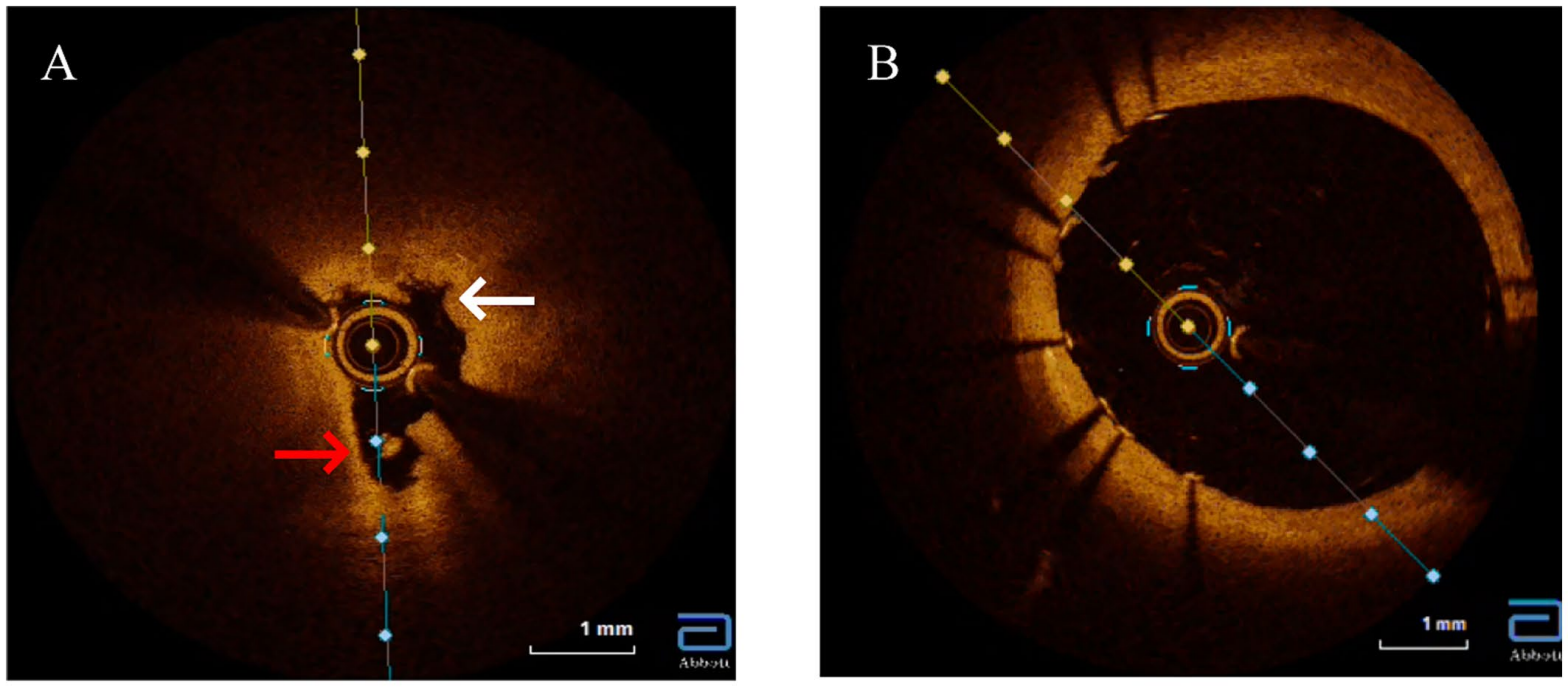
Optical coherence tomography (OCT) imaging of the left main ostium. **(A)** Baseline OCT reveals features pathognomonic for syphilitic vasculitis, including severe luminal narrowing, marked disruption of the medial layer (white arrows), obliterated vasa vasorum appearing as signal-poor voids, and diffuse adventitial microcalcifications (red arrows). Crucially, key features of atherosclerosis, such as a lipid core or a distinct fibrous cap, are absent. **(B)** Post-intervention, showing a well-apposed stent and restoration of the vessel lumen.

Given the constellation of ostial-predominant coronary disease, aortic aneurysm, and the profound discordance between angiographic severity and the objective lack of atherosclerosis (CACS = 0, normal CIMT, absence of traditional risk factors), an alternative etiology such as aortitis was strongly suspected. The positive TPPA test, indicating past or current *Treponema pallidum* infection, made cardiovascular syphilis the leading diagnostic consideration. To confirm or exclude this suspicion and to guide the optimal intervention, intravascular OCT was performed. Recognizing OCT’s unique capability to evaluate plaque morphology, composition, and stenosis etiology while assessing PCI risks, we opted for OCT-guided PCI rather than coronary artery bypass grafting (CABG) due to the focal ostial involvement and Rentrop grade 2 collateral circulation. OCT revealed severe ostial stenosis with medial layer disintegration, hyporeflective adventitial microcalcifications, and vasa vasorum obliteration (Figure 4A). Figure 4B showed stent implantation after PCI treatment. These features distinguished syphilitic vasculitis from atherosclerosis (which typically shows lipid-rich plaques) or Takayasu arteritis (diffuse intimal thickening).

Following successful percutaneous coronary intervention of the left main ostium with a 3.5 × 12 mm drug-eluting stent, the patient was started on dual antiplatelet therapy. Crucially, a definitive course of antibiotic therapy for cardiovascular syphilis was initiated, consisting of intramuscular benzathine penicillin G (2.4 million units weekly for 3 weeks), in accordance with standard guidelines. At the 6-month follow-up, the patient remained free of angina. A repeat echocardiogram demonstrated a notable improvement in left ventricular function, with the ejection fraction increasing from 29 to 35%. The severe functional mitral regurgitation and moderate aortic regurgitation secondary to the dilated aortic root remained unchanged. Following a comprehensive heart team discussion, and considering the patient’s significant symptomatic improvement and modest recovery of LVEF, a consensus was reached to continue guideline-directed medical therapy and close clinical surveillance, while deferring surgical intervention for the valvular disease at that time. A follow-up computed tomography angiogram confirmed the stable diameter of the previously identified thoracic aortic aneurysm.

## Discussion

Cardiovascular syphilis represents a late-stage complication of *Treponema pallidum* infection that continues to pose significant diagnostic challenges in contemporary practice (4). Typically manifesting 10–30 years after initial exposure, this condition predominantly affects the aorta, coronary ostia, and valvular structures, with classic presentations including aortitis, aortic regurgitation, and aneurysmal dilation (5). Our case illustrates a classic diagnostic dilemma of cardiovascular syphilis: proximal coronary stenosis. This dilemma was further compounded by serological ambiguity—a non-reactive RPR despite a positive TPPA—a pattern characteristic of late-stage disease due to immunological exhaustion (6). Given this ambiguity, our first critical step was to rule out alternative diagnoses, particularly atherosclerosis. In such clinically ambiguous scenarios, OCT emerges as a crucial diagnostic tool, providing detailed microstructural characterization when histopathological confirmation is unavailable (7). The OCT findings in our patient, including medial layer disruption, hyporeflective adventitial microcalcifications, and vasa vasorum obliteration, created a distinctive morphological signature differentiating syphilitic involvement from atherosclerotic disease or idiopathic aortitis. These features contrast sharply with the lipid-rich plaques with fibrous caps characteristic of atherosclerosis or the concentric laminar inflammation seen in giant cell arteritis, instead demonstrating fragmented elastic laminae and peri-adventitial scarring consistent with chronic ischemic injury from endarteritis obliterans (8). The absence of lipid pools or thrombus on OCT argued against acute plaque rupture, while the ostio-proximal localization with Rentrop grade 2 collateralization supported a slowly progressive inflammatory stenosis process (9). This case unequivocally underscores the limitation of relying solely on RPR in late-stage syphilis. A non-reactive RPR does not exclude active vasculitis, as evidenced by the positive TPPA and, crucially, the definitive OCT findings. This discrepancy, potentially reflecting the prozone phenomenon or a serofast state, underscores the necessity of a high clinical suspicion and the use of adjunctive imaging in such cases.

It is important to acknowledge the technical considerations of performing OCT at the coronary ostium and ostio-proximal segments. Achieving transient blood clearance in this location adjacent to the high-flow aortic root can be challenging. However, in this case, the critical need to determine the etiology of the stenosis (syphilitic vasculitis vs. atypical atherosclerosis) justified the use of OCT, as it provides unparalleled microstructural resolution. With meticulous catheter engagement and contrast flushing techniques, we successfully obtained diagnostic image quality. This underscores that while OCT offers unique diagnostic insights in such complex cases, its application in ostio-proximal lesions requires significant operator expertise.

The diagnostic value of OCT extends to its ability to differentiate syphilitic vascular involvement from other vasculitides (10). While Takayasu arteritis typically presents with diffuse intimal thickening and homogeneous signal intensity, syphilitic lesions demonstrate irregular medial hyporeflectivity. Similarly, IgG4-related aortitis primarily shows adventitial fibrosis without the microcalcifications characteristic of syphilis, and atherosclerotic lesions exhibit features like thin-cap fibroatheromas and necrotic cores that are absent in syphilitic vasculopathy (11). Based on these observations, we propose three OCT hallmarks of cardiovascular syphilis: focal medial disintegration, adventitial microcalcifications, and obliterated vasa vasorum—though these findings require validation in larger patient cohorts. The OCT findings were pivotal, as they provided the definitive morphological evidence that confirmed our suspicion after we had systematically excluded atherosclerosis. Indeed, the most critical step in our diagnostic process was this systematic exclusion. Several key pieces of evidence converged to make atherosclerosis an unlikely cause for the patient’s complete left main ostial occlusion. First, the patient lacked major traditional risk factors, including smoking, hypertension, and diabetes. Second, and most powerfully, the coronary artery calcium score (CACS) was zero. CACS is a highly sensitive marker for the presence of coronary atherosclerosis, and a score of zero confers an extremely low probability of obstructive atherosclerotic disease. The severe, non-calcified ostial lesion in our patient is therefore highly atypical for an atherosclerotic origin. Third, the absence of atherosclerosis was a systemic finding, supported by a normal carotid intima-media thickness and the lack of plaques in other major arteries. Finally, the lesion’s location—perfectly confined to the coronary ostium, adjacent to a dilated aortic root—is a classic hallmark of syphilitic aortitis, which primarily affects the aortic media and adventitia, leading to secondary ostial narrowing. In contrast, atherosclerotic lesions are typically more diffuse and located distal to the ostium within the coronary artery itself (Table 1).

**Table 1:** Differentiating features of vasculopathies on OCT.

Feature	Syphilitic vasculitis	Atherosclerosis	Takayasu arteritis	Key references
Primary layer affected	Media and adventitia	Intima	Intima and media	(6, 7)
Intimal thickening	Fibrous thickening, typically lipid-poor	Lipid-rich, fibrous cap, necrotic core	Homogeneous, laminar, fibrous	(6, 7, 12)
Medial structure	Disrupted with fragmented elastic laminae	Generally preserved or thinned by plaque	Frequently thickened and fibrotic	(6, 7)
Vasa vasorum	Prominently obliterated (endarteritis obliterans)	Intraplaque neovascularization	Periadventitial inflammation	(6, 7)
Calcification	Adventitial microcalcifications (hyporeflective)	Intimal, nodular or sheet-like (hyperreflective)	Typically absent	(6, 7)
Lumen shape	Irregular, “keyhole” configuration	Eccentric or concentric narrowing	Concentric, “macaroni-like” narrowing	(6, 7)

From a therapeutic perspective, our case underscores several critical considerations (12). While coronary artery bypass grafting remains the preferred approach for diffuse aortic involvement, isolated ostial lesions may be amenable to OCT-guided percutaneous coronary intervention. However, the persistent infection risk for stent restenosis necessitates concomitant antibiotic therapy with benzathine penicillin G (2.4 MU weekly for 3 weeks) and long-term surveillance. The decision to administer penicillin in this case of non-reactive RPR is firmly supported by established guidelines for the management of late-stage syphilis. The primary goal of antibiotic therapy in cardiovascular syphilis is to halt the progression of the infectious vasculitis and prevent further structural damage. Treatment is indicated regardless of the RPR titer, as a non-reactive result in this context is a recognized serological pattern, often reflecting a “serofast state” due to late-stage immunological exhaustion rather than absence of active infection. Consequently, treatment efficacy should be assessed through clinical evaluation and serial imaging to monitor for stability of aortic dimensions, rather than relying on serological titers. This case also serves as a cautionary example of the consequences of untreated syphilis, as our patient’s prior RPR positivity (titer 1:16) without treatment likely contributed to the development of these cardiovascular complications, emphasizing the importance of appropriate follow-up and treatment even in asymptomatic cases (13).

Several important limitations must be acknowledged when interpreting these findings. The invasive nature of OCT and the technical challenges of aortic imaging limit its utility as a first-line screening tool, and the procedure itself carries inherent risks that must be carefully weighed against potential diagnostic benefits. Furthermore, the etiology of the patient’s severe left ventricular dysfunction was likely multifactorial, arising from the combined effects of chronic ischemia due to coronary ostial stenosis and volume overload secondary to long-standing syphilitic valvulopathy. Although the improvement in LVEF after revascularization suggests a reversible component to the myocardial dysfunction, the absence of myocardial viability assessment with cardiac magnetic resonance imaging (MRI) represents a limitation, as it precludes a definitive delineation of the relative contributions of reversible functional impairment versus irreversible fibrotic remodeling to the overall systolic dysfunction. While our OCT findings correlate well with historical histopathological descriptions of syphilitic aortitis, the absence of tissue confirmation in this clinical case introduces some diagnostic uncertainty. Non-invasive alternatives such as FDG-PET/CT may serve as complementary imaging modalities, particularly for initial evaluation (14). Most importantly, the OCT features we identified as characteristic of cardiovascular syphilis require validation in larger, prospective studies to establish their sensitivity and specificity. Despite these limitations, this case provides compelling evidence for the role of advanced imaging in diagnosing challenging cases of cardiovascular syphilis, particularly when serological results are ambiguous. It underscores the need for clinicians to maintain a high index of suspicion for syphilitic vasculitis in patients with unexplained coronary ostial lesions, regardless of RPR serostatus, as early recognition and appropriate treatment can significantly alter the disease course. Future research should focus on further characterizing the imaging hallmarks of cardiovascular syphilis and developing standardized protocols for incorporating advanced imaging into diagnostic algorithms for these complex cases.

## Conclusion

In RPR-negative cardiovascular syphilis, OCT provides crucial diagnostic information when serology is inconclusive. Our findings support incorporating OCT into the diagnostic algorithm for suspected late-stage syphilitic vasculitis, particularly in serologically ambiguous cases. Given the potential for false-negative results due to the prozone phenomenon or rare seronegativity in late-stage syphilis, greater emphasis should be placed on clinical manifestations, OCT and other imaging modalities (e.g., angiography, CT/MRI), and histopathological findings when suspicion remains high. Early antibiotic therapy combined with vascular intervention, when indicated, is critical to halting disease progression. Close follow-up, including serial RPR testing and aortic imaging, is essential to monitor treatment response and disease stability. This case advocates for a multimodal diagnostic approach (OCT + angiography + serology + biopsy if needed) in atypical aortitis. Future studies should explore OCT’s predictive value for treatment response in tertiary syphilis and optimize strategies for seronegative cases.

## Data Availability

The original contributions presented in the study are included in the article/supplementary material, further inquiries can be directed to the corresponding authors.
